# Head and Neck Gross Tumor Volume Automatic Segmentation Using PocketNet

**DOI:** 10.1007/978-3-031-83274-1_19

**Published:** 2025-03-03

**Authors:** Awj Twam, Adrian Celaya, Evan Lim, Khaled Elsayes, David Fuentes, Tucker Netherton

**Affiliations:** 1 Department of Imaging Physics, The University of Texas MD Anderson Cancer Center, Houston, TX, USA; 2 Institute of Data Science in Oncology, The University of Texas MD Anderson Cancer Center, Houston, TX, USA; 3 Department of Abdominal Imaging, Division of Diagnostic Imaging, The University of Texas MD Anderson Cancer Center, Houston, TX, USA; 4 Department of Radiation Physics, The University of Texas MD Anderson Cancer Center, Houston, TX, USA

**Keywords:** Head and Neck Cancer, Gross Tumor Volume, Automated Segmentation, Convolutional Neural Networks

## Abstract

Head and neck cancer (HNC) represents a significant global health burden, often requiring complex treatment strategies, including surgery, chemotherapy, and radiation therapy. Accurate delineation of tumor volumes is critical for effective treatment, particularly in MR-guided interventions, where soft tissue contrast enhances visualization of tumor boundaries. Manual segmentation of gross tumor volumes (GTV) is labor intensive, time-consuming and prone to variability, motivating the development of automated segmentation techniques. Convolutional neural networks (CNNs) have emerged as powerful tools in this task, offering significant improvements in speed and consistency. In this study, we participated as Team Pocket in Task 1 of the HNTS-MRG 2024 Grand Challenge, which focuses on the segmentation of gross tumor volumes of the primary tumor (GTVp) and the nodal tumor (GTVn) in pre-radiotherapy MR images for HNC. We evaluated the application of PocketNet, a lightweight CNN architecture, for this task. Results for the final test phase of the challenge show that PocketNet achieved an aggregated Dice Sorensen Coefficient (DSCagg) of 0.808 for GTVn and 0.732 for GTVp, with an overall mean performance of 0.77. These findings demonstrate the potential of PocketNet as an efficient and accurate solution for automated tumor segmentation in MR-guided HNC treatment workflows, with opportunities for further optimization to enhance performance.

## 1 Introduction

Head and neck cancer (HNC) is among the most prevalent cancers worldwide, with over 500,000 new cases diagnosed annually [1]. Treatment strategies for HNC include radiation therapy (RT) as a critical component of a multimodal approach. Essential to RT treatment planning is the routine contouring of gross tumor volumes (GTVs) and organs at risk. Accurate delineation of tumor volumes is critical for effective treatment, particularly in MR-guided interventions, where soft tissue contrast enhances visualization of tumor boundaries [2]. However, manual contouring of these structures is both time and labor intensive and associated with known interobserver variability that can impact treatment outcomes [3, 4]. The development of automated tumor segmentation techniques using deep learning offers the potential to enhance the accuracy, reproducibility, and efficiency of tumor delineation.

Historically, traditional methods for tumor segmentation relied on atlas-based approaches or semi-automated tools that required manual adjustments and were limited by anatomical variability in HNC [5]. With the advent of deep learning, convolutional neural networks (CNNs) have emerged as the standard for automated medical image segmentation. State-of-the-art CNNs, such as U-Net and its variants, have demonstrated exceptional performance in segmenting tumors and organs from medical images [6]. However, these architectures often require extensive computational resources, limiting their accessibility and scalability in clinical settings. To address these limitations, we explored the application of PocketNet, a lightweight CNN architecture designed to reduce computational overhead while maintaining segmentation accuracy [7].

The Grand Challenge is a prominent platform designed to host competitions in medical imaging analysis, promoting advancements in machine learning. The HNTS-MRG24 challenge targeted the segmentation of GTV in HNC using MRI. For this study, we participated in Task 1 of the HNTS-MRG24 challenge, which focuses on the autosegmentation of GTVs from pre-radiotherapy (pre-RT) MR images and evaluated the performance of PocketNet for segmenting GTV in HNC patients.

## 2 Methods

### 2.1 Imaging Data

The data set used in this study was provided by the HNTS-MRG 2024 Challenge, which consisted of MR images of 150 pre-RT cases for Task 1 segmentation. These images included manually contoured masks of primary tumor (GTVp) and nodal tumor (GTVn), serving as the ground truth for training and validation.

### 2.2 Image Processing

The preprocessing pipeline from the Medical Imaging Segmentation Toolkit (MIST) was used to prepare the data. This pipeline processes NIfTI files and outputs reoriented, resampled, windowed, and normalized NumPy arrays. Pre-RT images were prepared using a patch size of 256 × 256 × 64 and a pixel spacing of 0.5 × 0.5 × 1.2 mm. The patch size was derived from the median resampled image size by selecting the nearest power of two less than or equal to each dimension, constrained by a maximum patch size. Initial target spacing is anisotropic with the maximum and minimum spacing along the lowest resolution axis [8]. All images were reoriented to right-anterior-inferior (RAI) to ensure uniformity Table 1.

### 2.3 Model Architecture

PocketNet is a lightweight, deep learning model based on the U-Net architecture. Unlike traditional CNNs, which double the number of feature maps at lower resolutions, PocketNet maintains a constant number of feature maps across all resolution levels. With the use of PocketNet, this results in faster training and inference times while simultaneously lowering memory usage and requirements [7, 8].

**Fig. 1.**
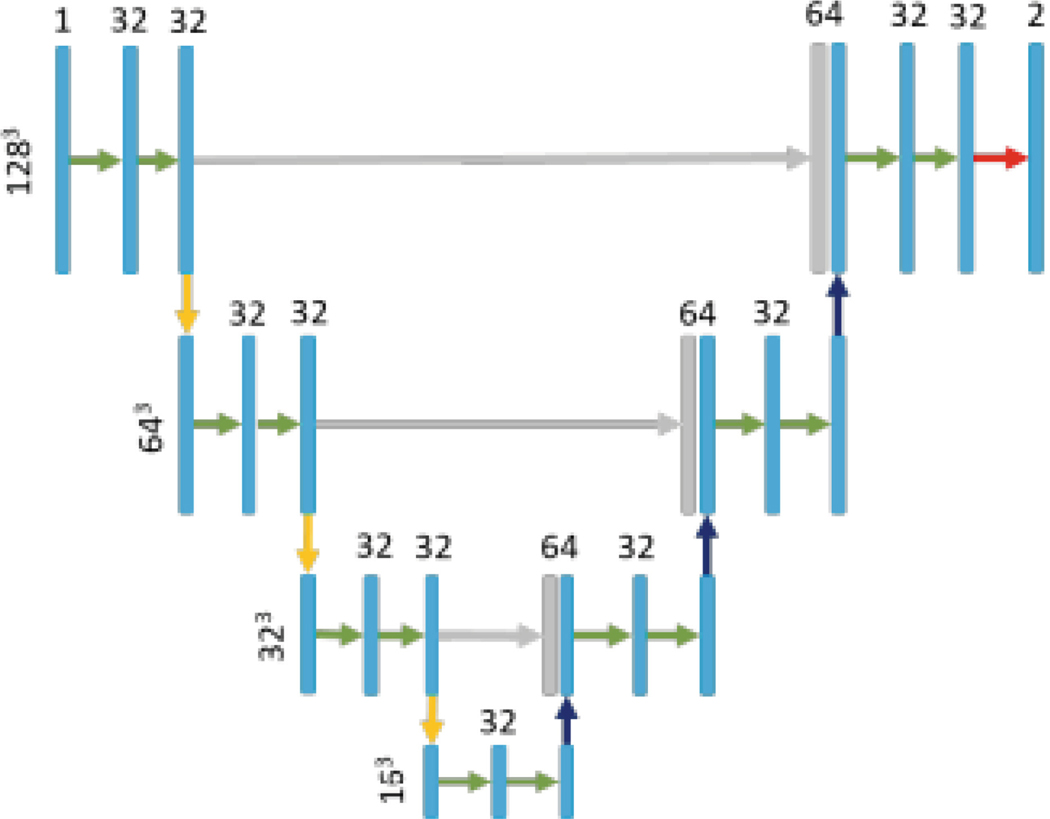
PocketNet architecture

Similar to a U-Net structure, our network is constructed from a composition of convolution and down sampling operations that extract features along a contracting path. An expanding path consists of convolution and up-sampling operations with ‘long skip’ connections to integrate features from the corresponding down sampling operations. Four resolution levels are used. At a given resolution, the feature-maps of all preceding layers are used as inputs, and its own feature maps are used as inputs into all subsequent layers. The key difference in our architecture is that the number of layers is not heuristically increased at each resolution. Each convolutional operation uses a 3×3 kernel size and is followed by a batch normalization and a ReLU activation function Fig. 1.

### 2.4 Model Implementation

The model was trained using an Nvidia Quadro RTX 8000 graphical processing unit (GPU) with a batch size equal to the number of patients per fold. A five-fold cross validation was conducted to ensure robust and generalizable results. For each fold, the network was trained for 1,000 epochs. During training, network weights were determined using the Adam optimizer. Additionally, automatic mixed precision was enabled to optimize memory usage and speed. The loss function combined Dice with categorical cross-entropy to ensure effective segmentation of both GTVp and GTVn. A constant learning rate of 0.0003 was used by default. Postprocessing included selecting the largest connected component from the predicted mask to reduce noise. To assess the model’s performance, two primary outcome metrics were used: the Dice Similarity Coefficient (DSC), which measures the overlap between predicted and ground truth segmentations, and the 95^th^ percentile Hausdorff distance (Haus95), which provides insight into the boundary accuracy of segmentations.

## 3 Results

### 3.1 Quantitative Evaluation

The performance of the PocketNet model for segmentation gross tumor volumes for HNC was evaluated using two key metrics: DSC and Haus95 for both primary tumor and nodal tumor. Table 2 below provides a summary of the statistics from training on 150 pre-RT MRIs, which includes the conventional DSC metrics.

The PocketNet model achieved the following segmentation performance for the final test phase (50 patients): 81% for GTVn and 73% for GTVp with an overall mean of 77%.

**Fig. 2.**
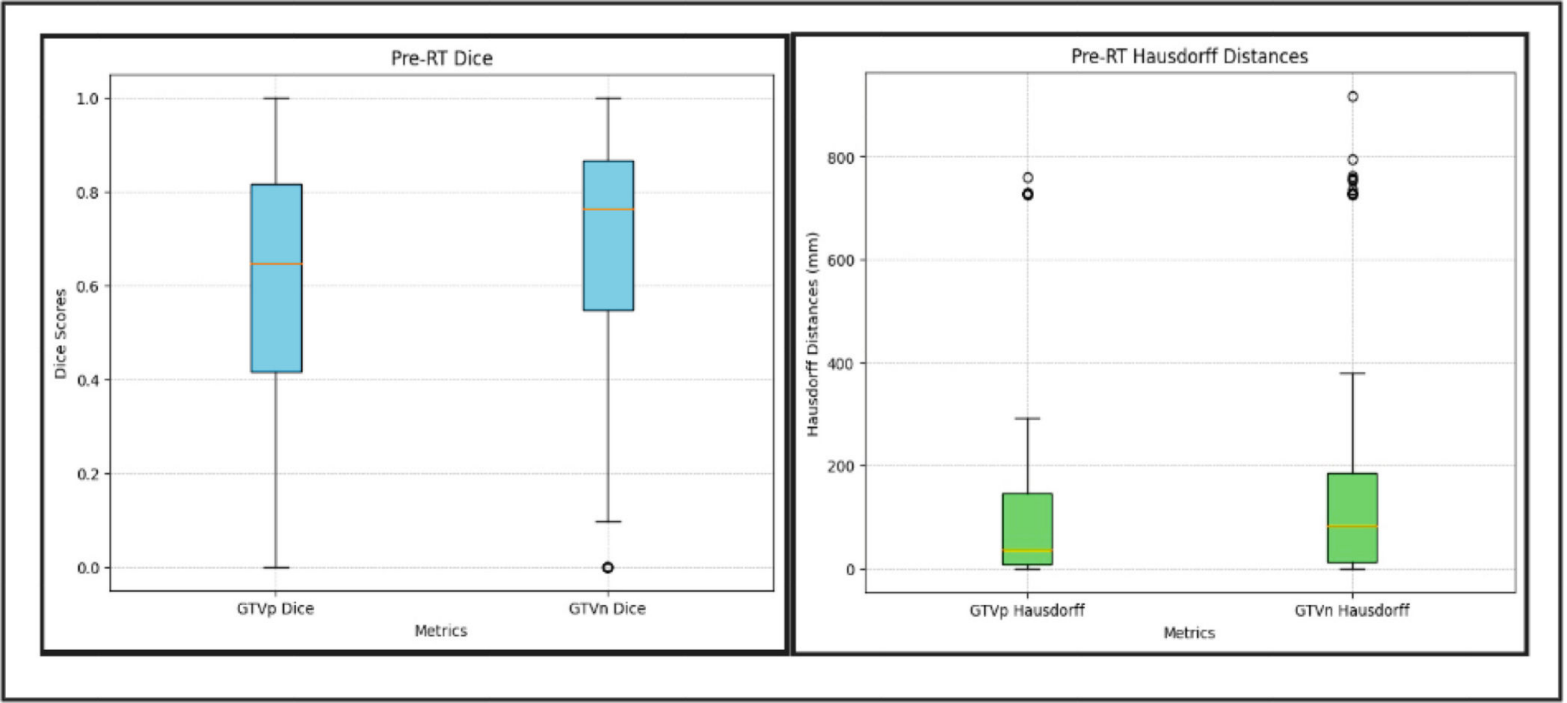
Box and whisker plots of conventional DSC and Haus95 metrics for Pre-RT

Figure 2 presents the conventional DSC and Haus95 metrics in the form of box and whisker plots. PocketNet, when trained via five-fold cross validation, achieved a mean DSC of 55% for GTVp and 64% for GTVn with a standard deviation (Std) of 0.314 for GTVp and 0.299 for GTVn. The distribution of DSC and Haus95 values can be visualized through the box and whisker plots. GTVp showed a wider distribution with whiskers extending lower compared to GTVn, which reflected a greater variability in performance on tumors. For GTVp, the median DSC is 0.648 and 0.763 for GTVn. GTVn displayed one outlier below the lower whisker, suggesting a base where the model struggled, likely due to atypical nodal morphology, smaller tumor size, and/or poor boundary contrast. The overall consistency for GTVn, with a median DSC of 0.763, underscores the model’s general robustness for nodal tumor segmentation. The mean Haus95 for GTVp was 130.139 mm and 173.818 mm for GTVn with a Std of 212.402 for GTVp and 238.689 for GTVn. For GTVp, 50% of the error values lie between 9.12 and 145.99 mm, while for GTVn, the range is between 12.28 mm and 185.64 mm. For both GTVp and GTVn, the larger upper quartile range reflected the difficulty the model faced in some cases, particularly when tumors are harder to delineate.

**Fig. 3.**
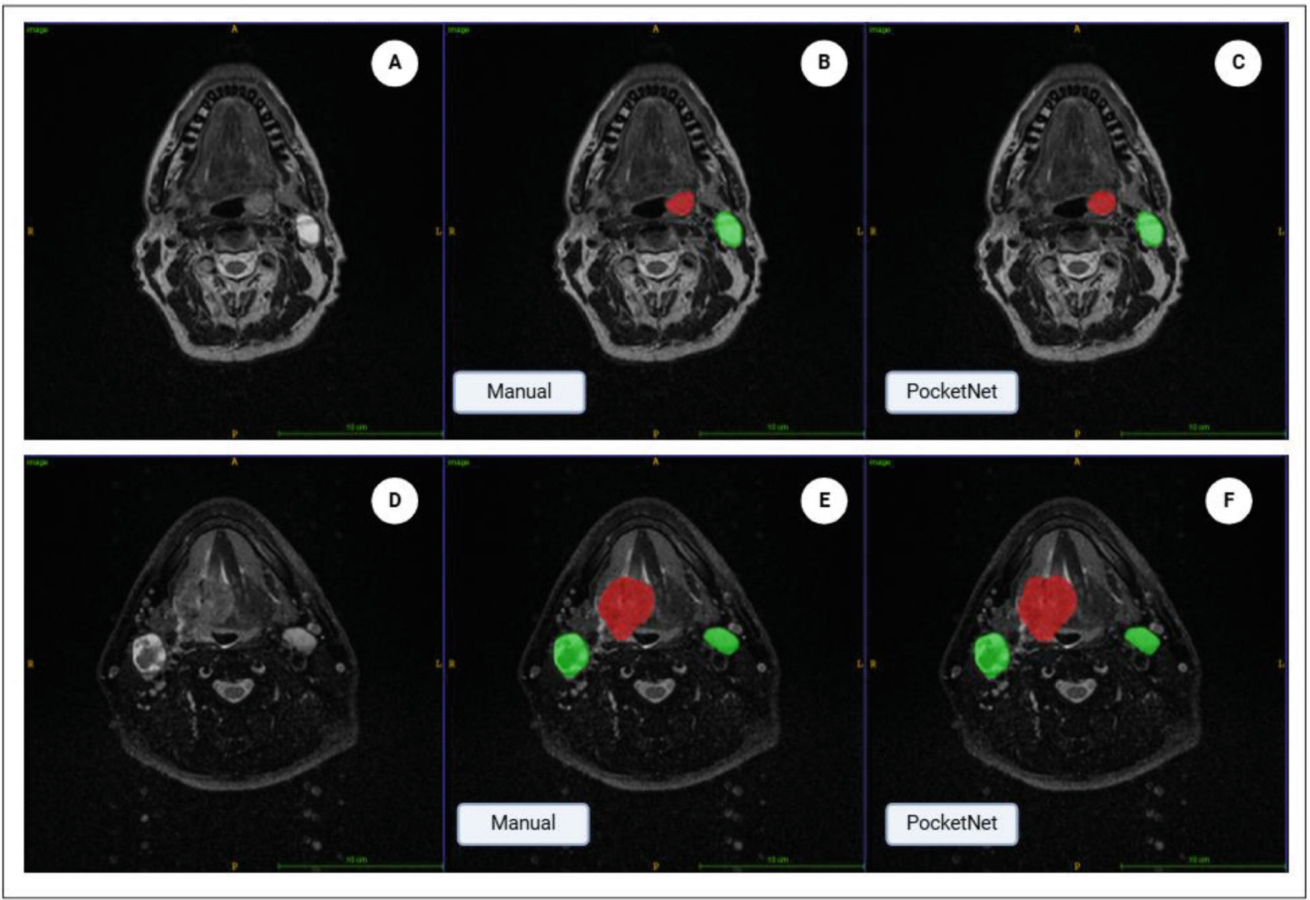
Manual and automated segmentation in two patients. A) Axial MRI of Case 1. B) Case 1 manual segmentation of GTVp (red) and GTVn (green). C) Case 1 Automated segmentation of GTVp (red) and GTVn (green). D) Axial MRI of Case 2. E) Case 2 manual segmentation of GTVp (red) and GTVn (green). F) Case 2 Automated segmentation of GTVp (red) and GTVn (green). The manual and automated tumor contours demonstrate good agreement (GTVp DSC = 0.88, 0.90 and GTVn DSC = 0.91,0.896).\CFO{}

**Fig. 4.**
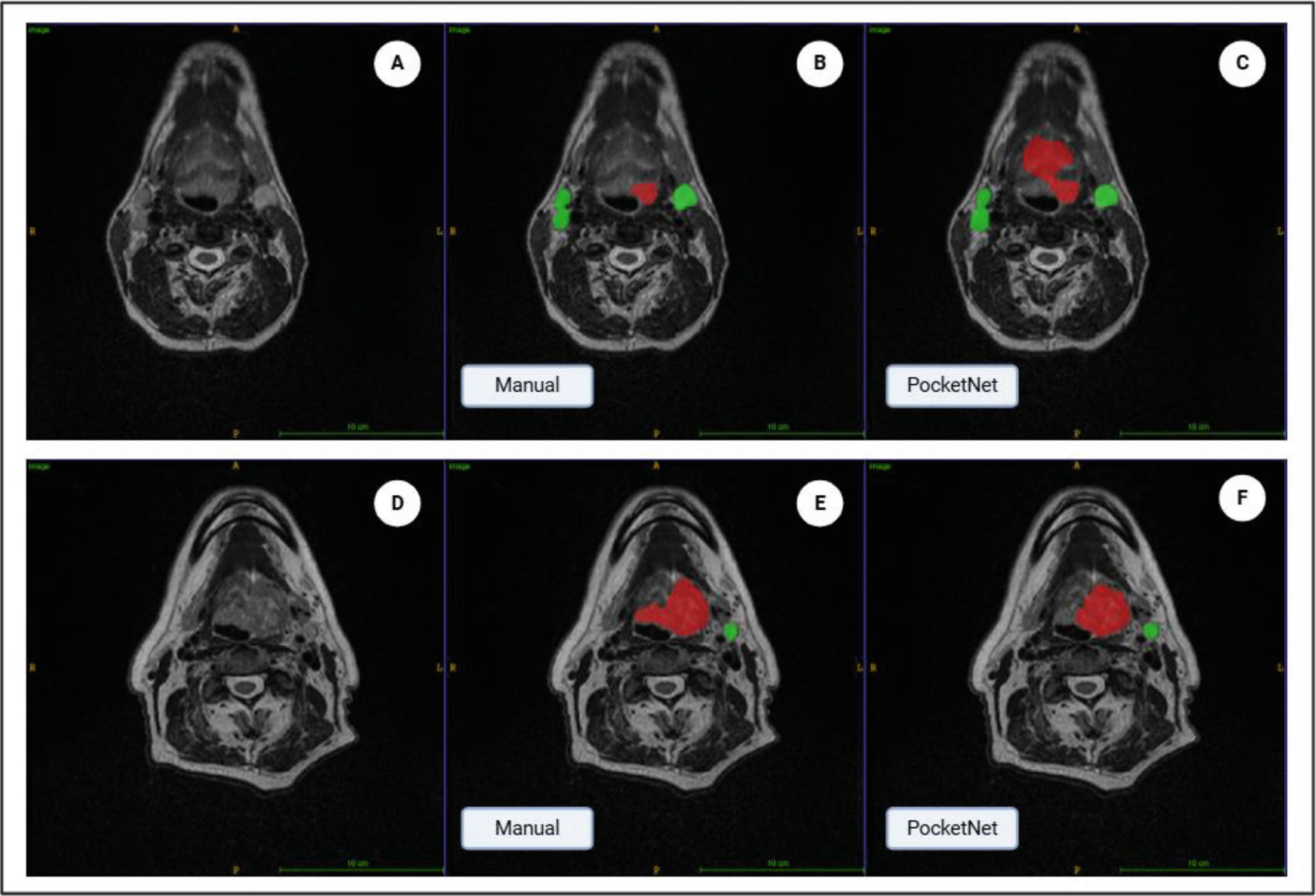
Visual results of segmentation in two patients. A) Axial MRI of Case 3. B) Case 3 manual segmentation of GTVp (red) and GTVn (green). C) Case 3 automated seg-mentation of GTVp (red) and GTVn (green). D) Axial MRI of Case 4. E) Case 4 manual segmentation of GTVp (red) and GTVn (green). F) Case 4 automated segmentation of GTVp (red) and GTVn (green). The segmentations demonstrate poor agreement of gross tumor volumes with a DSC of 0.51 for GTVp and 0.78 for GTVn for Case 3 and 0.75 DSC for GTVp and 0.58 for GTVn for Case 4.\CFO{}

Figures 3 and 4 illustrate example cases of segmentation. Figure 3 presents two cases of segmentation with good agreement. Case 1 had DSC scores of 0.88 for GTVp and 0.91 for GTVn, while Case 2 below it had DSC values of 0.90 for GTVp and 0.896 for GTVn. In contrast to Fig. 3, Fig. 4 presents two cases of segmentation with adequate to poor agreement. Case 3 in Fig. 4 had a DSC value of 0.51 for GTVp and 0.78 for GTVn and Case 4 below it had a DSC of 0.75 for GTVp and 0.58 for GTVn.

### 3.2 Qualitative Evaluation

To further understand the performance of the automated segmentation model, we evaluated the results by comparing the DSC coefficients between the ground truth and the automated contours, identifying key factors that may have contributed to discrepancies in segmentation accuracy. The variation in Dice values across the two cases in Fig. 4 is primarily due to the model’s overestimation or underestimation of the tumor volumes relative to the ground truth. In Case 3, the model underestimated the GTVp volume resulting in a lower DSC of 0.51. In Case 4, the GTVp segmentation model showed a higher degree of overlap (DSC of 0.75), but autosegmentation of GTVn overestimated portions of the nodal tumor. Tumor shapes in HNC can vary significantly. The lower Dice scores, in particular the GTVp in Case 3 of Fig. 4, may be due to the model’s difficulty in capturing accurately the full extent of the tumor, especially in areas where the tumor had fewer clear boundaries. This challenge is compounded by the DSC inherent bias toward volume, as smaller regions of interest contribute less to the overlap metric.

## 4 Discussion

PocketNet demonstrated strong performance in Task 1 (pre-RT) of the HNTS-MRG 2024 challenge, achieving mean DSCagg scores of 0.808 for GTVn and 0.732 for GTVp in the final phase of testing. These results are comparable to those from similar segmentation challenges. For instance, in the HEKTOR challenge at MICCAI 2022, the winning model achieved DSCagg values of 0.801 for GTVp and 0.775 for GTVn [9]. PocketNet’s comparable performance highlights its potential as an efficient segmentation tool with reduced computational requirements, though opportunities for further improvement remain.

A recent study on CT-only GTV segmentation for palliative head and neck radiotherapy highlights the complexities of automated contouring in anatomically challenging regions. Using the nnU-Net architecture, the study evaluated five approaches across two datasets of non-contrast and contrast-enhanced CT scans. Median DSC ranged from 0.6 to 0.7, with Haus95 between 14.7 mm and 19.7 mm. While the performance was competitive, the study emphasized the challenges of delineating nodal involvement and poor tissue soft contrast in CT-only workflows [10]. The CT-only segmentation results align with the challenges addressed by PocketNet, reinforcing the importance of developing models that pereform well in single-modality imaging.

The advantages of multi-modal imaging are well-documented in segmentation tasks. For example, a study using 3D CNNs to automate the delineation of GTV for HNC compared single-modality (CT) and multi-modality (CT + PET and CT + MRI) data. The results showed that multi-modality CNNs performed better, achieving a mean DSC of 69% for GTVp and 79% for GTVn [11]. These results, while slightly lower for GTVp compared to PocketNet, underscore the advantages of incorporating multi-modal imaging, particularly for anatomically complex regions like the head and neck. A separate study conducted by Ren et al. demonstrated that combining CT, PET, and MRI for head and neck tumor segmentation using a 3D Residual U-Net achieved a Dice coefficient of up to 0.74 when utilizing ensemble bi-modal combinations, with PET inclusion shown to be particularly impactful [12]. Similarly, Wahid et al. found that incorporating multiple parametric MRI (mpMRI) channels (e.g., T2 + T1) significantly improved segmentation performance for oropharyngeal primary tumors, achieving a Dice coefficient of 0.73 and also demonstrated non-inferior results compared to ground truth segmentations in a Turing test with clinical experts, further validating the potential of multi-modality imaging in enhancing segmentation accuracy [13].

While this study was focused solely on MR imaging for pre-RT segmentation, the methodology could be extended to mid-RT segmentation tasks (Task 2 of the HNTS-MRG 2024 challenge). Mid-RT segmentation prediction is critical for adaptive radiotherapy, where tumor shrinkage or progression during treatment necessitates updated contours. While we did not evaluate PocketNet on Task 2, future work could explore its application in this context. Additionally, we are conducting an ablation study using the pre-RT data to better understand the contribution of individual components of PocketNet’s architecture to its overall performance. This analysis aims to identify key features that most significantly impact segmentation accuracy, guiding future model refinements. By optimizing the model’s architecture and fine-tuning its parameters, we hope to enhance segmentation accuracy.

Overall, PocketNet represents a promising solution for automated segmentation of GTVp and GTVn in MR images of HNC patients. Its lightweight design, combined with competitive segmentation accuracy, positions it as a robust tool for clinical workflows.

## 5 Conclusion

Manual contouring of GTV and organs-at-risk can lead to interobserver variability and impact treatment outcomes for HNC patients. Our proposed deep learning segmentation model, PocketNet, with its compact architecture and optimizations for light GPU memory usage and fast inference times, addresses key challenges in automating the segmentation of anatomical regions of interest for HNC radiation treatment planning. These results demonstrate that PocketNet achieved an average DSCagg of 0.808 for GTVn and 0.732 for GTVp for the final test phase of Task 1 in the HNTS-MRG 2024 challenge. Future work employing this model is anticipated to further refine and validate this approach for automated segmentation.

## Figures and Tables

**Table 1.** Preprocessing of data

Dimension	Value
**Patch Size**	256 × 256 × 64
**Pixel Spacing**	0.5 × 0.5 × 1.2 mm

**Table 2.** Summary statistics of Dice and Haus95 metrics

	GTVp Dice	GTVn Dice	GTVp Haus95	GTVn Haus95
Mean	0.549	0.641	130.139	173.818
Std	0.314	0.300	212.402	238.689
25th Percentile	0.416	0.546	9.124	12.283
Median	0.648	0.763	37.184	82.717
75th Percentile	0.815	0.867	145.986	185.642
